# Modelling phenotypes, variants and pathomechanisms of syndromic diseases in different systems

**DOI:** 10.1515/medgen-2024-2020

**Published:** 2024-06-06

**Authors:** Anne Gregor, Christiane Zweier

**Affiliations:** University of Bern Department of Human Genetics Inselspital Bern 3010 Bern Switzerland; Department of Human Genetics Inselspital Bern 3010 Bern Switzerland

**Keywords:** hiPSC-based models, rodent models, *Drosophila melanogaster*, zebrafish, disease modeling

## Abstract

In this review we describe different model organisms and systems that are commonly used to study syndromic disorders. Different use cases in modeling diseases, underlying pathomechanisms and specific effects of certain variants are elucidated. We also highlight advantages and limitations of different systems. Models discussed include budding yeast, the nematode worm, the fruit fly, the frog, zebrafish, mice and human cell-based systems.

## Introduction

With the recent advances of sequencing technologies and therefore growing numbers of identified novel diseases and (candidate) disease genes, also the utilization of suitable models has become more and more important. Reasons and aims for utilizing model systems or organisms in syndromic diseases include the following: a) to obtain a better understanding of both the phenotypic expression and the underlying pathomechanisms as well as to characterize genotype-phenotype correlations, b) to confirm novel disease genes, c) to validate variants of unknown significance, d) to obtain platforms for studying potential therapeutic approaches in a pre-clinical setting. Depending on the disease and the research questions and aims, a variety of model systems and organisms is available. The following overview outlines commonly used models with a description of application areas, advantages and limitations.

## Budding yeast (*Saccharomyces cerevisiae*)

The budding yeast *Saccharomyces cerevisiae* is a single-cell eukaryotic microorganism known as baker’s yeast. It is a widely used model organism in functional genetics studies as it is relatively cheap, easy to grow and to genetically manipulate due to its haploid status. Its smaller genome and fewer gene duplications allow studying biological and biochemical functions and pathways in a simplified system compared to complex eukaryotes, but limits testing of functions that are specific for higher order organisms [1,2]. 31.5 % of human rare disease genes are conserved in yeast [3].

*S. cerevisiae* has been successfully utilized to investigate pathomechanisms for various disease groups such as neurodegenerative disorders including Huntington’s and Alzheimer’s disease [1] or mitochondria-related disorders [4]. For example, by observing dysfunction of iron homeostasis and mitochondria upon knockdown of the yeast frataxin, important insights into its function and thus into the pathomechanisms of Friedreich ataxia were obtained [5].

Furthermore, *S. cerevisiae* can be a powerful tool to confirm pathogenicity of single candidate variants, as recently been demonstrated for a *de novo* missense variant in KIF21A in an individual with developmental delay, neurodegenerative decline, microcephaly and myelination abnormalities [6]. Loss of a conserved glutamate residue within the switch II motif of KIF21A (and yeast Cin8/Kip3) resulted in kinesin motor activity indicated by impaired yeast proliferation [6].

*S. cerevisiae* also represents an ideal first tier drug screening platform, particularly for disease groups with highly conserved pathways and mechanisms as for example for mitochondrial disorders [4]. In the so-called drug drop test, mutant yeast cells with defects in oxidative growth are spread on plates and covered with sterile filters containing different compounds. After several days, growth behaviour of mutant cells is compared to a positive control, indicating either rescue, toxic or lacking effects. This allows high throughput testing of >10 000 compounds and identified several rescuing substances in yeast models for different mitochondrial disorders as reviewed by [4].

## Nematode worm (*Caenorhabditis elegans*)

*C. elegans* is a transparent, free-living soil nematode with a length of 1 mm and a diameter of approximately 80 µm. It is easy to grow on a bacterial diet and has a short life cycle. There are only 959 somatic cells in the adult hermaphrodite, 302 of which are neurons. Despite its rudimentary organization, many cell types with complex functions in mammals such as muscle cells, neurons, gut and excretory cells are present and identifiable in *C. elegans* [7]. Phenotypes that can be assessed include viability, locomotion, feeding, reproduction, responses to stimuli and learning and adaption [3]. 66 % of human rare disease genes are conserved in the worm [3]. Loss of function or haploinsufficiency of specific genes can be modeled by targeted knockdown via RNA interference approaches by feeding worms with dsRNA expressing bacteria [8]. More recently, CRISPR/Cas9 technology has been utilized for complete knockout or precise genome editing [9]. By gonadal microinjection of DNA, transgenic animals can be generated [10], either overexpressing a worm gene or ectopically a human gene. Such gain-of-function or toxicity models have been particularly used to investigate pathomechanisms of neurodegenerative diseases such as amyotrophic lateral sclerosis and Huntington’s, Alzheimer’s and Parkinson’s disease [11]. Apart from that, *C. elegans* has been proven to be a valuable model in investigating pathomechanisms of mitochondrial diseases [12], but also of other disorders such as fibrillinopathies [13], spinal muscular atrophy, polycystic kidney diseases and dystrophinopathies [7].

Furthermore, *C. elegans* can be utilized for rapid variant validation and for drug screening [14]. Approximately 13 % of variants in humans already have a corresponding variant in *C. elegans* [15]. Also genotype-phenotype correlations can be determined, as for example a missense variant in ion channel NALCN identified in a child with a particularly, severe lethal clinical presentation of CLIFAHDD syndrome (Congenital contractures of the limbs and face, hypotonia, and developmental delay, MIM: 616266) was shown to result in a gain-of-function effect in *C. elegans* with hypercontraction and uncoordinated movement. Other missense variants in NALCN identified in affected individuals with CLIFAHDD resulted not only in gain-of-function but also in loss-of-function effects [16]. Furthermore, variants in non-conserved amino-acids can be tested in whole-gene humanized animal models [14]. For this, the native gene is replaced by the human orthologue. If the human gene rescues the phenotype associated with loss of the worm gene, most human coding variants can be studies by assessing rescue effects compared to the wildtype. For example, human *PTEN* and *KLC4* have been successfully introduced into *C. elegans*, respectively, and variant pathogenicity was investigated [17,18].

## Fruit fly *(Drosophila melanogaster*)

*Drosophila melanogaster* has been one of the most important model organisms in biological and biomedical research within the last 100 years. It has a very short life cycle and is easy and inexpensive to maintain. Moreover, a huge variety of transgenic strains and tools for genetic manipulation are publicly available, and many established protocols for phenotype assessment exist [19]. 73.1 % of human rare disease genes are conserved in the fly [3].

Approaches to manipulate gene expression in a targeted way include the UAS/GAL4 system for ubiquitous or tissue specific knockdown or overexpression [20] as well as CRISPR/Cas9 related approaches for complete knockout or knock-in of specific variants [21]. Apart from investigating the effect of reduced (modeling loss of function and/or haploinsufficiency) or increased (modeling gain of function) dosage of a particular gene, there is a multitude of approaches to investigate the effect of specific missense variants. Patient-derived variants can be introduced in the fly ortholog if the amino acid is conserved, or rescue experiments can be performed with overexpressing the wildtype and mutant fly gene in a fly strain deficient for the endogenous gene. Variants affecting non-conserved amino acids can be assessed in humanized rescue approaches. For this, wildtype and mutant human gene constructs can be expressed in a fly deficient background to perform rescue experiments. Alternatively, these constructs can be overexpressed in an unaltered fly background to assess possible toxic effects. For example, overexpression of a human transgenic construct carrying one of five missense variants identified in *PPFIA3* in individuals with a neurodevelopmental disorder showed that variants in the N-terminal coiled-coil domain result in stronger phenotypes than variants in the C-terminal region. Additionally, in contrast to the human wildtype, overexpression of three of the variant transgenic constructs failed to rescue embryonic lethality caused by homozygous loss of its orthologue, suggesting they are dominant negative loss-of-function alleles [22] Therefore, such experiments do not only allow conclusions on the pathogenicity of a particular variant but also indicate loss-of-function, gain-of-function or dominant negative effects [3,23].

Assessable and quantifiable phenotypes in *Drosophila* include amongst others viability, morphology (e.g. wings, eyes, bristles, brain, neurons, synapses) as well as habituation and complex learning and behavior. *Drosophila* is therefore an established and very broadly used model to investigate neurodevelopmental and neurodegenerative disorders. Its utilization has contributed to validating and obtaining pathomechanistic insights into numerous neurodevelopmental disorders (NDDs), both on gene and on variant level [24,25]. Using easily quantifiable phenotypes (e.g. wing morphology) as phenologs, *Drosophila* also allows systematic, large scale screens to characterize common molecular links between NDD genes [26] or to identify functional links between genes implicated in clinically overlapping disorders by genetic interaction experiments [27]. Apart from its prominent role in modelling neurodevelopmental disorders, *Drosophila* is also utilized as a model for e.g. kidney [28] or cardiac diseases [29] and mitochondrial disorders [30].

*Drosophila* also can serve as a platform for drug screening or identification. The first hint that mGluR antagonists might pharmacologically rescue Fragile-X syndrome associated symptoms came from a *Drosophila* study [31].

## Frog (*Xenopus tropicalis* and *Xenopus laevis*)

The western clawed frog *Xenopus tropicalis* is a diploid species, while the South African clawed frog *Xenopus laevis* contains a duplicated set of genes and chromosomes. *Xenopus* is a rapid, cost effective, high-throughput vertebrate organism to model particularly developmental defects and congenital organ malformations [32]. 91.4 % of human rare disease genes are conserved in *X. tropicalis* [3]. One of the advantages of *Xenopus* is the easy accessibility to its eggs and embryos which allows intracellular microinjections and thus precise and organ specific manipulation up to the late tadpole stages. Unique among animal models is the possibility of one-sided injections with the contralateral side serving as an internal control [32]. Gene expression in *Xenopus* embryos can be manipulated by injecting mRNA for gain of function or morpholino oligonucleotides for loss of function, respectively. Furthermore, CRISPR/Cas9 has been used to induce either a complete knockout of a gene of interest or to induce specific genomic changes [33].

As early tadpoles are transparent, thus allowing assessment of organogenesis by light microscopy, *Xenopus* has been broadly used to model congenital cardiac defects, heterotaxy, primary ciliary dyskinesis and kidney defects [32]. For example, targeting two ciliary chondrodysplasia loci (ift80 and ift172) by CRISPR/Cas9 resulted in severe limb deformities, polydactyly and cystic kidney in froglets, closely matching the phenotype in humans with skeletal ciliopathies [34]. However, also other specific human phenotypes can be reproduced. For example, knockdown of the *Xenopus* ortholog of *PYCR1* resulted in skin hypoplasia and blistering of the tadpole skin, thus resembling the human phenotype of cutis laxa caused by bi-allelic variants in *PYCR1* [35]. Also complex developmental syndromes and neurocristopathies affecting several organs and presenting with craniofacial abnormalities such as CHARGE and Kabuki syndromes [36] or RASopathies [37] have been successfully modeled in *Xenopus*.

## Zebrafish (*Danio rerio*)

Zebrafish are small, 3–4 cm long freshwater fish with a life span of 2 years and a generation time of 3 months. They present a vertebrate model with ex-utero development, making it easily feasible to observe all stages of development, especially given the fact that embryos are transparent, and all cells are visible from the outside until early larval stages. It is cost effective with limited space requirements for maintenance, and can produce large numbers of eggs [38]. Studies on embryos in the first 5 days post fertilization are not regulated, only after that zebrafish larvae will be considered experimental animals under Animal Welfare legislation [39]. Zebrafish has a high degree of genetic, anatomical and physiological similarity to humans with orthologs for 94.5 % of human rare disease genes [3,40]. The existence of an efficient genetic toolbox and the ease of genome manipulation through various techniques (morpholinos, ZF-nucleases, TALENs, CRISPR/Cas9) has also contributed to its success as a model organism [41].

Zebrafish has been widely used to study effects of loss-of-gene-function either through morpholino studies or more recently through knockouts using CRISPR/Cas9 genome editing. Additionally, effects of specific variants can be tested either through overexpression of human wildtype or mutant versions for the gene of interest in wildtype fish or through rescue experiments upon gene knockdown/out using morpholinos or CRISPR/Cas9 and simultaneous co-injection of the wildtype and mutant human gene [42]. Studies can be both performed during early embryonic development and in adult fish. A wide variety of organ systems have been studied making zebrafish a very suitable model to study syndromic disorders. Studied diseases include epilepsy, behavioural anomalies, kidney, heart defects, skeletal malformations and retinal and hearing defects. Zebrafish has a high capability for tissue regeneration, which has been especially studied in the heart. Ease of substance administration through the water has also allowed for drug screenings in zebrafish, at least for water soluble substances [43].

A prototype example of disease modelling in zebrafish are ciliopathies and especially Joubert syndrome, a multisystem disorder with common symptoms, among others being cerebellar and brainstem malformations, retinal degeneration, cystic kidney disease and polydactyly [44–46]. A high level of conservation is present both for specialized cilia and morphologically for the retina [47], the pronephros as a simplified human nephron [48] and bones [49]. Knockouts of many ciliopathy-related genes in zebrafish display cystic kidneys, retinal dystrophy and spinal curvature in larvae [50] and also show functional consequences of knockouts at the organismal level such as impaired visual function assessed in the oculo-kinetic response [51]. Several available tools furthermore have allowed to study mechanisms of how different ciliary genes work and have provided crucial insights into ciliary function and dysfunction [50].

As with any model system, it does come with several limitations. Zebrafish has many duplicated paralogs due to an early genome duplication [40], which can create difficulties in generating full gene knockouts, as it often requires targeting of multiple paralogs in parallel [52]. Generation of genetic knock-ins using CRISPR/Cas9 genome editing still has a rather low efficiency in zebrafish, thereby hindering studies of specific mutations in zebrafish embryos and larvae and requiring at least F2 generations of fish [52]. While zebrafish is a good model for many organ systems, differences in brain anatomy limit its use in neuroscience [53]. However, more recently, functional similarities have been recognized, and zebrafish has been used to model for example behavioural abnormalities and epilepsies [54]. Furthermore, loss of fertility upon inbreeding of fish makes maintenance of mutants challenging, and only a limited number of mutant strains is deeply characterized and maintained.

## Mouse (Mus musculus)

Mouse models are the most widely used mammalian model organisms in rare disorder research and are making up around 60 % of research animals to date [55]. They are small, relatively cost-effective to maintain with an efficient reproduction cycle and share many similarities in anatomy and physiology with humans with 97.8 % of rare human disease genes conserved [3,56]. Large scale projects have generated knockout mouse models for many human disease genes (e.g. international knockout mouse consortium) [57], and the international mouse phenotyping consortium has performed systematic phenotyping for a large number of mouse lines [58]. Additionally, genome editing techniques such as CRISPR/Cas9 have also allowed to generate mouse models for specific disease-associated variants [59].

Mouse models have been particularly useful to model limb- and skeletal malformations, often recapitulating the human phenotype well. Here, also models for non-coding variation e.g. affecting chromatin topology have provided striking insights into disease pathology of various human disorders [60]. Mouse models have also been frequently used to study neurodevelopmental syndromic disorders, such as for example Angelman syndrome, where genomic organization is conserved in the mouse and neurological phenotypes recapitulate human phenotypes [61]. Those mouse models have then also been used for pre-clinical studies of gene-replacement and antisense-oligonucleotide (ASO) therapies [62,63].

However, challenges remain regarding high variability between different mouse mutants for the same disorder in different inbred backgrounds [64], which can confound results and impair transfer of knowledge. Furthermore, mouse models do not necessarily mimic human disease pathology accurately [65], which has led cofounding results and contributed to a lack of translatability of pre-clinical results to human clinical trials, e.g. in Fragile-X syndrome [66]. Additionally, societal and regulatory pressures have called for reduction of animal experiments, wherever possible [67], also increasing the search for other, possibly human-related disease and pre-clinical models.

## Other mammals

While mice present the most used rodent model, it has some limits regarding specific functions or organs. For some phenotypes that require a better reflection of the human situation, other mammalian models are utilized.

As many genetic mouse models of Huntington’s disease do not show the typical neurodegeneration known from human individuals, transgenic rats may be used. They either express a human/rat combined fragment of the HTT gene or the full-length HTT genomic sequence with 97 CAG/CAA repeats and all regulatory regions. These rats show an early or later onset progressive neurodegeneration mimicking the human disease course [68].

For modelling cardiac diseases such as long-QT syndrome, short-QT syndrome and hypertrophic cardiomyopathy, transgenic rabbits expressing human pathogenic variants are used. Their electrophysiological, mechanical and structural cardiac characteristics resemble the human situation better than small rodent mouse models [69]. For the generation of transgenic rabbits, pronuclear microinjection, the sleeping beauty transposon system and genome-editing methods such as zinc finger nuclease (ZNF), transcription activator-like effector nuclease (TALEN) and CRISPR/Cas9 are used [69].

Larger animals such as dogs, pigs and primates are only rarely used as models as they are less accessible to genetic manipulation and due to ethical and societal reasons. As an example, a genetically modified porcine model for Duchenne muscular dystrophy carriers presented with hyperCKemia, abnormal dystrophin expression patterns in skeletal and cardiac muscles, histopathological signs of muscle degeneration, myocardial lesions in adulthood and sporadic death [70]. Furthermore, “naturally” occurring variants in larger animals can reflect particular clinical aspects known from human individuals quite well, e.g. for connective tissue disorders. As reviewed in [71], dogs and cats with Ehlers-Danlos syndrome show thin and hyperextensible skin, and cattle with Marfan syndrome have aortic dilatation, ocular abnormalities and skeletal involvement.

## Cell-based models

A variety of different cell-based systems has been used for functional analysis aiding variant classification, for understanding pathomechanisms and for preclinical drug design and testing. Especially human cell-based systems can display important advantages over animal models and can overcome challenges in non-translatability of results from animals to humans, especially in drug discovery efforts. The recent advances in the development of 3D cellular systems have made them a very attractive tool to study diseases not only on a cellular level, but also on an organ and even organismal level. Different systems with different levels of complexity are described below.

### Immortalized cell lines

Testing of specific effects of (missense) variants via overexpression studies can be done largely independent of the affected organ systems and can therefore be performed in immortalized cell lines in the absence of disease relevant patient samples/tissue. Common cell lines used for such experiments are HEK293, HeLa, U2OS or neuroblastoma cell lines. Advantages of such approaches include that they are relatively quick and cost-effective, that cell lines are easy to manipulate with common transient transfection technologies, and that they often provide specific information regarding mechanistic effects of specific variants that are more difficult to obtain in some animal models. As an example, in epilepsy research, HEK293 cells are frequently used to heterologously express specific ion channels in the absence of endogenous channel activity to test effects of specific patient-related point mutations [72,73]. Using this approach, it could be deciphered how different variants in SCN2A that lead to different clinical phenotypes have different effects on SCN2A function [72]. Limitations of these overexpression studies include that modelling does not occur in the tissue of interest and therefore specific effects may be masked due to the lack of necessary context-specific co-factors or interaction partners. Additionally, careful selection of experimental controls is necessary as many variants may not express well and functional read-outs of assays may not be appropriate [74].

### Patient-derived primary cells (fibroblasts)

Patient-derived fibroblasts represent a very suitable disease model and have been used for several disorders to uncover abrogated pathways or biochemical functions in a patient-related context and to aid prioritization and characterization of potential disease-related variants. Amendable disorders include metabolic and mitochondrial disorders, while diseases affecting specific tissues (e.g. neurodevelopmental disorders or neuromuscular disorders), may not be modelled well in fibroblasts. However, it has been shown for example for Rett syndrome, that abrogated biochemical properties are well conserved in fibroblasts [75]. Additionally, the potential to reprogram fibroblasts into hiPSCs opens many opportunities for their use to model disorders tissue-specifically, which is replacing experimental work on fibroblast models for various disorders, such as for example neurodevelopmental disorders (see below). Patient-derived fibroblasts have the advantage that they are relatively easy to obtain through a skin biopsy, and that they can be cultured, so that amount of material to be used in experiments is not strongly limited. Patient fibroblasts have for example been used as a source of RNA to combine RNA-Seq analysis with whole exome/genome sequencing to aid diagnostics. This has successfully been done for mitochondrial disorders, where expression changes due to deep-intronic splice variants improved identification pathogenic variants [76]. Choice of correct tissue to perform RNA-Seq analysis on may limit the use of fibroblasts for many disorders, e.g. neurodevelopmental or neuromuscular disorders, but has been implemented in clinical diagnostics [77]. Another way of aiding variant classification and providing evidence for variant pathogenicity from fibroblasts are rescue experiments. For this, following identification of a measurable phenotype in patient fibroblasts (e.g. enzymatic readout, defect in OXPHOS, biochemical or microscopic assay), rescue experiments can be performed with wildtype or mutant transgenes [2]. Depending on the assay, transgene expression is either performed through transient transfection for assays assessing single cells, or stable transduction using (lenti)-viral particles and subsequent antibiotic selection when high levels of transduction efficiency are required for the assay (e.g. enzymatic readouts). Possible limitations come from expression of the transgene from an exogenous promotor at non-physiological levels, which can either mask rescue effects if expression is too high or too low or can have detrimental effects on the cells in the long-term. One way to circumvent this issue can be the use of inducible promoters (e.g. tetracycline-responsive element containing promoters). This approach has for example been applied successfully multiple time for various mitochondrial or metabolic disorders [2]. Generally, it can be difficult to obtain proper, matched controls, especially when affected individuals are very young. It also has to be considered that mutational load of fibroblast samples can vary depending on the area of skin that biopsy was obtained from, which may impact assay results [78,79].

**Figure 1:**
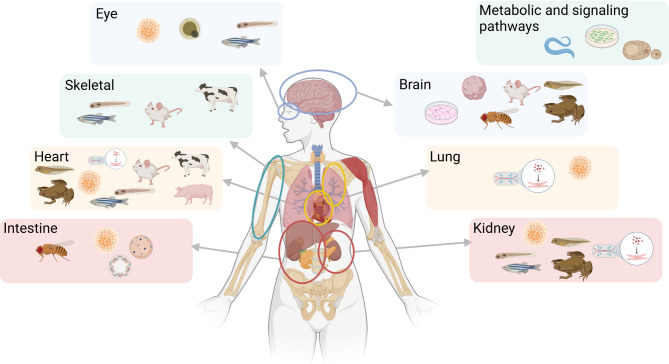
** Schematic overview over different model systems and tissues that are commonly modelled.** Created with BioRender.com.

### iPSC-based cellular models in 2D

With the development of somatic cell reprogramming technologies, it became feasible to reprogram patient cells into human pluripotent stem cells (hiPSC) [80]. HiPSCs can theoretically be reprogrammed from all actively dividing somatic cells, most commonly this is done using skin fibroblasts. Other starting tissues that have been used successfully include peripheral or umbilical cord blood cells, where CD34+ are isolated, keratinocytes from hair and tubular epithelial cells isolated from urine samples [81]. They can then be differentiated into different tissues of interest from all three germ layers and have been used among others to model different types of neurons, retinal cells, endothelial cells, intestinal cells, hepatocytes, cardiomyocytes, renal cells and lung epithelial cells [82].

Depending on the questions to be answered, it may be more suitable to either use patient-derived cells for reprogramming or to introduce patient-specific variants using CRISPR/Cas9 in control cells. Advantages of the use of patient-derived cells reprogrammed to hiPSCs include that the genetic landscape of the patient including potential disease modifiers is conserved. However, genomic instability and epigenetic memories during reprogramming [83] in addition to a lack of matching controls may increase variability in experiments due to different background, age, sex, ethnicity, or different reprogramming techniques [84]. In contrast, when introducing variants with CRISPR, isogenic not targeted controls are available from the same experiment, therefore less variability due to different backgrounds may be expected. A possible, but cost- and labour-intensive work-around is to use patient-derived hiPSCs and remove disease causing variants using CRISPR/Cas9 as isogenic experimental controls. Especially for modelling not strictly monogenic, oligogenic or complex disorders or disorders with reduced penetrance or variable expressivity, patient-derived models represent the full genetic complexity of the patient and are therefore ideally used.

**Table 1:** Overview over different model systems

Model organism	Model for	Modelled disorders	Genetic manipulation
**Yeast**	– disease pathways and simple functions – variant pathogenicity – drug screening	– mitochondrial and neurodegenerative disorders	– Transformation
***C. elegans***	– disease pathways and simple functions – variant pathogenicity	– neurodegenerative disorders	– RNA interference (soaking in or feeding dsRNA) – Microinjection for transgene expression – CRISPR for knockout (and specific variants)
***Drosophila melanogaster***	– development and function – variant pathogenicity and effects – pathomechanisms	– cardiac, kidney, nervous system diseases	– CRISPR for knockout (and specific variants) – UAS-GAL4 system for knockdown (RNAi) and overexpression
***Xenopus***	– organogenesis	– heart, kidney diseases	– Morpholinos and mRNA injection for knockdown and overexpression – CRISPR for knockout (and specific variants)
**Zebrafish**	– variant pathogenicity and effects	– Skeletal, eye, heart, kidney diseases	– CRISPR for knockout (and specific variants) – Morpholinos for knockdown – TALENs, transgene overexpression
**Mouse**	– phenotyping – pathomechanisms	– Skeletal, brain, heart diseases	– CRISPR for knockout and specific variants
**Cell-based modeling**	– variant pathogenicity and effects – pathomechanisms	– Eye, brain, heart, lung, intestine, kidney diseases	– CRISPR for knockout and specific variants – Transient and stable transfection for knockdown and overexpression

For syndromic disorders, disease-relevant hiPSC lines can be differentiated into different lineages and tissues to model different aspects of disorders. Examples include hiPSC-based models for Williams-Beuren syndrome, where differentiations into neural precursor cells and neurons showed electrophysiological dysregulation [85], and differentiation into smooth muscle cells recapitulated the vascular phenotype observed in patients [86]. In hiPSC-derived disease models for Bardet-Biedl syndrome, defects in insulin and leptin signalling in neurons related to obesity observed in patients [87] and abrogated ciliary structures and signalling in retinal sheets as might be expected in a ciliopathy [88] were observed.

HiPSC models have also been used successfully to study effects of combinations of therapeutic compounds. For instance, in cystic fibrosis research various hiPSC models differentiated into lung epithelial cells, pancreatic ductal epithelial cells and intestinal cells reflecting three of the major affected organs exist and were used to test different therapeutic strategies [89–91].

Limitations of hiPSC 2D models include lack of organismal context as cells are usually cultured in a monoculture not representative of the complex interplay of different cell types and extracellular matrix in different organs [92], therefore, non-cell autonomous pathomechanisms cannot be assessed in 2D [93].

### iPSC-based cellular models in 3D (organoids & organs on a chip)

To overcome limitations such as a lack of tissue context in 2D iPSC-based models, recent developments have made it possible to generate 3D models for many organs that have also allowed for important novel insights in modelling syndromic disorders, either through the formation of organoids or organs-on-a-chip [94]. Organoids are mostly self-organizing, grown in matrix and recapitulate some level of tissue architecture and function [95]. Organoids have been generated for several organs, including the brain, heart, lung, liver, pancreas, intestine, kidney and retina. For instance, cortical brain organoids were first used to model microcephaly and could show that premature neuronal differentiation was one of the hallmarks in these organoids [96]. In a model of renal phenotypes in Bardet-Biedl syndrome, normal morphology was observed in a 2D model, but spontaneous degeneration of tubular structures was found in an organoid model, highlighting the added value of 3D disease models to reflect pathogenesis [97].

Organs-on-a-chip are 3D microdevices that can be used to control tissue composition and can incorporate vascular perfusion [98]. Especially for organs like lung and heart, this can provide valuable information on physical forces that occur in living organs [94]. One example is the modelling of Barth syndrome associated cardiomyopathy, where mitochondrial dysfunction could be linked to specific contraction defects in an hiPSC-derived cardiomyocyte organ-on-a-chip and where it was shown that variants in the associated *TAZ* gene are necessary and sufficient to cause these defects through gene-replacement experiments [99].

Advantages of 3D model systems include that they recapitulate organ-level structure or function to some degree and can even be combined for multiple organs through generation of assembloids, e.g. fusion of cortical organoids and skeletal muscle spheroids to form 3D cortico-motor assembloids [100] or combinations of multiple organs-on-a-chip. Several challenges in the field remain such as high costs and labour-intensiveness, high heterogeneity and variability between different batches of organoids and even within batches, likely due to their self-organizing nature. Furthermore, organoid growth is often limited in size due to the lack of nutrients inside organoids once they reach a certain size, although many protocols are being developed to establish vascularized organoids to overcome this. Experimentally, it remains challenging to set up live, in-vivo experimental readouts from organoid cultures that are not endpoint measurements. For organs-on-a-chip, technical limitations remain in throughput, which remains low, and the need for manufacturing of custom-made chambers and devices [101].

With the use of patient-derived iPSCs, these models also present exciting new opportunities in disease modelling and as preclinical models in drug-testing overcoming some of the limitations in translatability that other non-human model systems present.
